# Gleanings in Science and Medicine

**Published:** 1893-09-30

**Authors:** 


### Gleanings in Science and Medicine.

Greenwich time has recently been adopted at Brussels as official measure, both within the precincts of the town, also its immediate suburbs. It is only to be expected that the regulation will extend throughout Belgium, to avoid the confusion following on conflicting evidence of clocks. At the Belgian capital the new measure was carried at the Hotel de Ville by universal acclaim.

---

The Russians have shown a great consideration for their tram-horses during the recent excessive heat in the large towns. In Odessa, men were stationed at the various termini of the tram routes, or midway on larger journeys, provided with buckets of ice-cold vinegar and water, with which to bathe the horses' heads. The operation took but a few seconds, but under it the animals seldom showed any kind of weariness. A horse dropping down from exhaustion is unknown in Russian towns during the summer season, and tram horses are all well kept and groomed. Indeed, our own highly civilized country could take a lesson with great advantage in both of these respects.

---

Two German physicians have arrived at the conclusion, independently of one another, that in many cases where persons have fallen victims to lightning, had artificial means of rescusitation to life been brought to bear on their behalf, the majority of these lives would have been saved. For the doctors contend that being struck by lightning is not necessarily fatal, in spite of appearances to the contrary. The means to restore life in this instance being the same as those which are employed to the bodies of persons rescued from drowning, and the promulagators of this new theory, whilst they point out the wide difference between the causes of the apparent suspension of life in each case, yet assert that both yield alike to the same restorative methods.

---

The verdict of the jury of scientists who have been voluntarily sitting on the case *re* "Scorpion Suicides" has been awaited with much interest by a race whose suicidal tendencies are of a more firmly established nature. The anecdote of the rattlesnake at the Lick observatory, which, upon being captured, took upon itself a form of deliverance from temporal captivity known amongst mortals under the name of suicide, has elicited a large amount of corroborating evidence on the same heading from various quarters, from persons who stand ready to trace a fresh analogy between themselves and the world immediately below them in the animal scale. Professor E. Ray Lankester gives his judgment on these cases of so called "scorpion suicide" from a rational and common sense standpoint, and whilst he asserts the non-lethal effect of a snake's poison on its own person, he effectively puts the case of snake self destruction out of court altogether.

---

The Australian musical profession have been brought face to face with the problem of how to meet the increasing demand in the Colonies for their services being gratuitously rendered in aid of hospitals and other charitable objects. The prevailing depression in trade has affected all classes of the community alike, and musicians have an undoubted right to defend themselves against a demand for their stock-in-trade, which, whilst entailing a pecuniary outlay on their part, brings with it no returns.

---

An original departure in tinned commodities, show itself in the French industry of icing milk. Milk is frozen, and placed in the block form into tins, which on the part of the purchaser requires to be melted previous to use. Being hermetically sealed the commodity thus iced preserves its form until it is required, when a minute's exposure to the sun's rays, or to the heat of the fire, is all that is necessary. It is not likely that this means of conserving the fluid will interfere with the industry which the Swiss originated, for this newer method possesses the disadvantage of necessitating a use at the same time of the whole contents of the tin, as when once unsolidified, it cannot be guaranteed to keep its freshness; at the same time milk thus preserved retains its original form and is not artificially sweetened.

---

The Federal Council of Switzerland are again at work with the difficult problem which has beset them regarding the increased consumption of alcohol in the country. They find that merely raising the price of the popular intoxicant known under the familiar name of "Schnapps" among the people, has availed little to lessen the demand for it; the peasantry preferring, apparently, to relinquish other necessaries of life rather than be deprived of the luxury of this decoction. This indulgence is affecting the country in a serious manner regarding the army list; it has been recently proved that over fifty per cent. of the young men who would be serving in the ranks are unfit to do so through physical deterioration, consequent on over-indulgence in this respect. At home we have no such statistics to go upon, but there are more deaths due to excessive drinking among English people than we are at all aware of, and the Registrar-General's returns convey to our mind no adequate representation of how matters stand in this regard. Medical certificates, so long as they are handed to relatives of the deceased, instead of going direct, under seal, to the local registrar's office, are only too likely to be found couched in terms of an equivocating nature, when the cause of death is one which leaves it open to the doctor to use a choice of terms. To give one instance amongst many, few practitioners are to be expected to be possessessed of sufficient moral courage to ascribe death as being due to a gin drinker's liver, when "liver complaint" would read more considerately to the feelings of those left behind.

